# Amputation Risk Factors in Severely Frostbitten Patients

**DOI:** 10.3390/ijerph16081351

**Published:** 2019-04-15

**Authors:** Anna Carceller, Casimiro Javierre, Martín Ríos, Ginés Viscor

**Affiliations:** 1 Secció de Fisiologia, Departament de Biologia Cel·lular, Fisiologia i Immunologia, Facultat de Biologia, Universitat de Barcelona, 08028 Barcelona, Spain; gviscor@ub.edu; 2 Medical Commission of the International Federation for Climbing and Mountaineering (UIAA MedCom), CH 3000 Bern, Switzerland; 3 Departament de Ciències Fisiològiques, Facultat de Medicina, Universitat de Barcelona, 08907 Barcelona, Spain; cjavierre@ub.edu; 4 Secció d’Estadística, Departament de Genètica, Estadística i Microbiologia, Facultat de Biologia, Universitat de Barcelona, 08028 Barcelona, Spain; mrios@ub.edu

**Keywords:** frostbite, risk factors, amputation, winter sports

## Abstract

In recent years, the incidence of frostbite has increased among healthy young adults who practice winter sports (skiing, mountaineering, ice climbing and technical climbing/alpinism) at both the professional and amateur levels. Moreover, given that the population most frequently affected is healthy and active, frostbite supposes a substantial interruption of their normal activity and in most cases is associated with long-term sequelae. It particularly has a higher impact when the affected person’s daily activities require exposure to cold environments, as either sports practices or work activities in which low temperatures are a constant (ski patrols, mountain guides, avalanche forecasters, workers in the cold chain, etc.). Clinical experience with humans shows a limited reversibility of injuries via potential tissue regeneration, which can be fostered with optimal medical management. Data were collected from 92 frostbitten patients in order to evaluate factors that represent a risk of amputation after severe frostbite. Mountain range, years of expertise in winter mountaineering, time elapsed before rewarming and especially altitude were the most important factors for a poor prognosis.

## 1. Introduction

Frostbite is a type of cold-related injury caused by the exposure of a part of the body to temperatures below the freezing point of the tissue, which is estimated to be −0.55 °C. Frostbite has increasing incidence as a non-occupational affliction in young healthy adults, linked to the increase in the popularity of winter mountain sports [1]. Cutaneous circulation plays an important role in thermoregulation by varying blood flow through peripheral structures in order to maintain core body temperature, which is essential for survival. In a cold environment, maximal vasoconstriction in hands and feet is reached when their temperature drops to 15 °C. This is followed by local protective cycles of vasodilation if cooling persists [2], but leads to progressive local ischaemia if exposure continues. Meanwhile, direct cellular injury caused by cold includes intra- and extracellular ice formation, structural alterations of cells and their components, and osmotic changes. The length of cold exposure and rapidity of freezing determine the magnitude and importance of these different pathological processes [3]. Frostbite is an injury that involves local ischaemia, cell injury and both local oedema and thrombotic events related to reperfusion of tissue damaged due to cold [4,5]. The microvascular impairment determines the potentially hypoxic tissues that can lead to necrotic areas [3]. According to the depth of the skin damage, necrosis can be so severe that it results in spontaneous or surgical amputation. Amputation is related to the severity of the injury, with the most severe injuries being more likely to result in non-viable tissue [6].

Numbness and coldness of the injured part count as secondary clinical signs and symptoms, after vasoconstriction and ischaemia, and are usually of little help for predicting prognosis before rewarming.

Thawing restores blood flow and induces congestion, inflammation and thrombosis in the injured endothelium [7,8], which may prompt erythrocyte extravasation due to failure of the vessel wall. Clinical manifestations include severe pain and macroscopic changes that can help to predict the extent of the injury [6]. 

Efforts have been made to establish risk factors for frostbite [9,10,11], as well as risk factors for amputation once the patient has reached in-hospital care [12,13]. However, little is known of the possibility of reducing amputation rates and improving the quality of injury management in the field once frostbite has already occurred. In this study, we aimed to determine which factors influence prognosis in established frostbite secondary to cold exposure in mountaineers, and what strategies to recommend in immediate care in order to achieve better injury outcomes.

## 2. Materials and Methods

We performed a retrospective study of data from 92 patients (74 men, 18 women) aged 33.1 ± 8.5 years, with 12.3 ± 9.5 years of mountaineering experience in winter conditions, who had recovered from acute frostbite injury regardless of the presence or absence of long-term sequelae. Patients (75% amateurs and 25% professionals) from different countries anonymously completed a survey once they had restarted their normal working or sporting activities. We collected the following data: gender, age, smoking habits, professional affiliation (if one existed), years of experience at activities over 3000 m in altitude, existence of previous preparation one year and five years before the accident, frostbite date, location (mountain and range), altitude, maximal altitude reached in the expedition, awareness of frostbite, modification of objectives, acute mountain sickness incidence, perception of fatigue previous to frostbite, affectation of decision making, specific causes of frostbite, time to reach first aid and first-aid post location. We also recorded details concerning the nature of the therapeutic measures received, recovery and rehabilitation and changes in habits or equipment; and on the existence of sensitivity alterations, sequelae and amputation. The single patient who suffered frostbite during a mountain rescue was excluded from the analysis, leaving a total of 91 subjects in the study. None of them did refreeze after rewarming. Chi-square, ANOVA with normality variables, Kruskal–Wallis and U-Mann–Whitney for non-parametric variables, and stepwise regression tests were performed to study interactions between variables and to check for risk factors. Data are presented as mean value and standard deviation. Statistical significance was considered when *p* < 0.05. After approval of the study by the local ethics committee, all the participants were informed of the objective of the study and freely gave their consent to participate.

## 3. Results

No differences were found with regard to age, gender, smoking habits or occupation between frostbitten patients needing surgical amputation (A = 68) and those who did not (NA = 23). The perception of fatigue prior to the frostbite incident was recorded on a scale ranging from 0 (absence) to 10 (maximal), suggesting a tendency towards correlation with the amputation group, although this did not reach statistical significance (6.9 ± 2.4 (A) vs. 6.0 ± 2.3 (NA); *p* = 0.083). 

We found statistically significant differences in the number of years of experience in winter mountaineering activities (10.8 ± 9.2 years for NA, 16.7 ± 9.5 years for A group; *p* < 0.001). The average altitude at which the frostbite occurred was 4850 ± 2400 m, with considerable differences between the groups (4210 ± 2210 m (NA) vs. 6760 ± 1910 m (A); *p* < 0.001), as well as in the maximum altitude reached during the ascent (4920 ± 3130 m (NA) vs 7190 ± 1860 m (A); *p* < 0.001).

**Figure 1:**
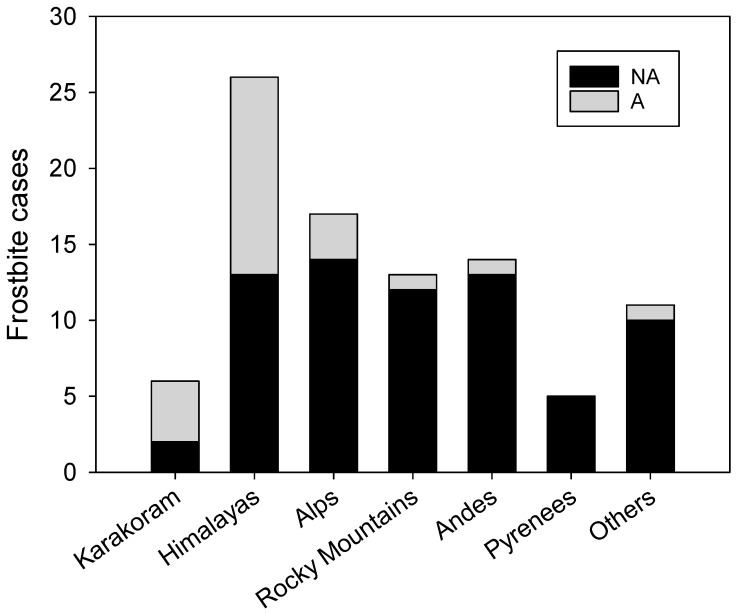
Occurrence of frostbite according to the main mountain ranges in our sample. Cases requiring amputation represented as grey bars stacked over those not requiring amputation denoted in black. The “Others” group of ranges include several mountains over 2000 m and Antarctica.

The time lapse between injury and first attention showed significant differences (23.5 ± 27.3 h for NA vs. 42.1 ± 31.6 h for A group; *p* = 0.003) as well as the mountain range in which the frostbite occurred (Figure 1), with the Himalayas and Karakoram showing the greatest incidences (*p* < 0.001). Only one case (Mt. Cook, New Zealand) of frostbite resulting in amputation was recorded outside of the Earth’s highest ranges. 

Capacity to make correct decisions seemed to be impaired in both frostbitten groups, 6.8 ± 2.5, although non-significant statistical differences were found.

**Figure 2:**
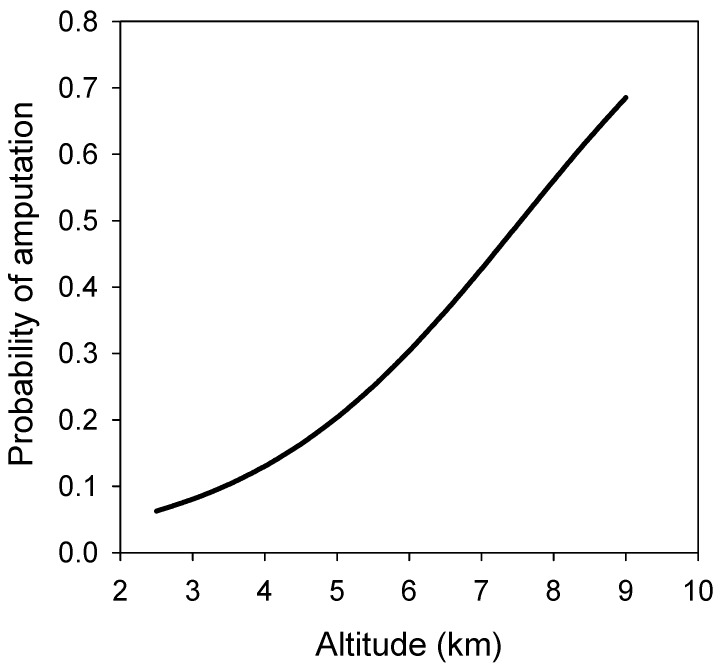
Relationship between probability of amputation after frostbite and altitude.

We developed a stepwise regression model [14] to establish the main risk factors for surgical amputation once severe frostbite occurs, considering the different variables that were included in the survey. We found that altitude had a very strong weight in our model, to the extent that the rest of the variables considered (including mountain range, years of experience and delay before first attention) were far less relevant for amputation prognosis as independent factors. However, these other variables are also strongly related to altitude, andafter maximum likelihood estimation regression analysis, altitude alone was a good predictor of amputation incidence. The probability of amputation after frostbite for any specific altitude, as yielded by this simplified mathematical model, is depicted in Figure 2.

In Table 1 we have listed the cut-off points of the above regression function with their corresponding values for the probability of error for prediction of amputation risk after frostbite occurrence.

Taking 0.55 as the index value for the prediction of amputation probability (in bold in Table 1), there is a 47.62% probability of making a correct prediction for amputation and a 92.45% probability of making a correct prediction for patients who will not undergo amputation. This is the cut-off point that suits to make the least error in what is most important for medical advice. Thus, we obtained the following formula, termed the amputation index:

(AI) = −4.04446 + 0.000535925 × altitude (expressed in metres).(1)

**Figure 3:**
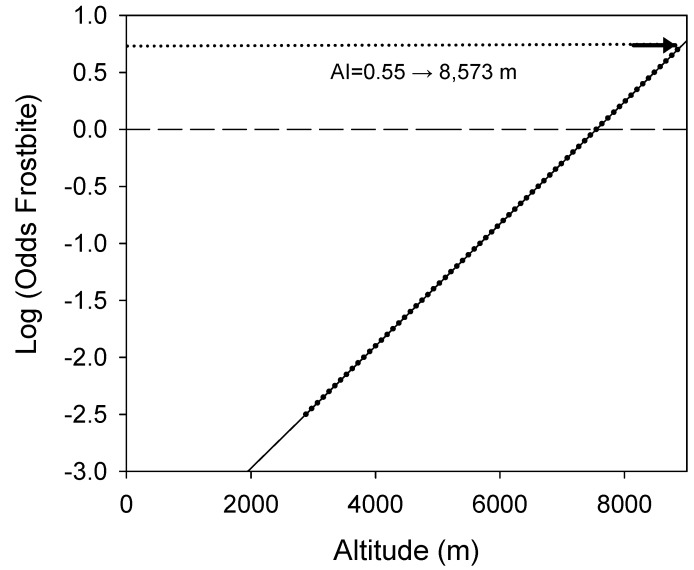
Amputation index in relation to altitude.

The results of this index can be interpreted as the 0 value representing a 50% probability of amputation if frostbite occurs, which corresponds to an altitude of 7547 m. As has been shown, the maximal value of our sample corresponds to an AI of 0.55, which corresponds to a 100% probability of amputation if severe frostbite occurs at altitudes over 8573 m (Figure 3). 

## 4. Discussion

According to our results, altitude is the most relevant amputation risk factor. This could be expected from the well-known lapse rate of a drop of approximately 1 °C every 150 metres of ascent (about 6.5 °C/1000 m), and the predisposition of individuals to experience peripheral vasoconstriction to maintain core temperaturein the cold environements. Haemoconcentration, dehydration, small vessel blockage and hypercoagulability resulting from a hypobaric environment may also play an important role [5,15]. Recent publications indicate that the additional effect of hypoxia on top of cold exposure results in a greater damage to microcirculation in animal models [16]. Acute hypoxia itself does not seem to have any relevant effect on tissue temperature [17]. The effects of acclimatization to altitude on this phenomenon are yet to be established, although laboratory observations suggest that it could worsen frostbite injuries due to the altered haemorheological behaviour [16]. In addition to this, hypoxia impairs rewarming responses of injured tissue [18]. Whether the causal mechanism of this finding is the additional effect of hypoxia on the severity of the injury due to vasoconstriction, the impairment of rewarming mechanisms, the increased difficulty for the subject to reach medical assistance and protection, or a combination of all factors is still unknown. It is worth noting that altitude was the strongest risk factor of the analysed variables, but the influence on altitude of other confounding factors in the field remain to be revealed. 

An increased number of years of experience at winter activities appeared to be a risk factor in our sample population. This is contrary to what could be expected, considering that experience in winter mountaineering may lead to an increase in knowledge of protection and prevention strategies against cold. It is worth noting that frostbitten patients in this study showed a high degree of expertise in mountaineering in winter conditions, averaging 12.3 years of preceding practice. Prior observations regarding risk and mountaineering experience indicate that climbing experience at high altitude does not have a positive influence on mortality. Furthermore, there is a positive association between mortality and the number of expeditions at high altitude [19,20]. In the case of avalanche accidents, an increase in mortality due to the repeated exposure to the hostile environment has been observed among more experienced and skilled subjects [21]. Our results suggest that experience per se should be considered a strong risk factor for suffering deep frostbite. This observation can be explained by the fact that experience may enable subjects to take part in activities that are more technical and performed at higher altitude. These may occur in more remote areas and may therefore have lower probability of receiving prompt medical attention. Finally, years of experience in winter mountaineering might imply a prior history of frostbite or alterations on distal peripheral circulation that have not been considered in the survey and might have an unknown influence on our results. 

Delay before receiving first medical attention for the frostbite injury after it was already produced was another strong risk factor for posterior amputation. This may be related to limitations in reaching medical installations in some mountain ranges (the Himalayas and Karakorum between them saw most of the severe frostbite leading to amputation in our study) and the lack of knowledge of frostbite first aid of the subjects involved. The average time until rewarming the injury was 23.6 h for those not requiring amputation, whereas this increased to 42 h for those who did require amputation. This observation is consistent with previous publications that suggest that the length of time for which tissue is exposed to cold is more damaging than the absolute temperature reached [4,22]. Different durations of freezing had different influences on blood circulation in frostbitten tissues, with the longest being the most harmful [16]. According to our results, time before first rapid rewarming of frostbite is a crucial factor for the risk of amputation, especially during the first 24 hours after frostbite occurs. The time to reach a clinical setting in order to receive advanced treatments (e.g., intravenous vasodilators or thrombolytic agents) was not considered in this study as none of the subjects received them. However, evidence suggests that reducing the time lapse before reaching an advanced medical post with possibilities of receiving more complex treatments such as thrombolytic factors and/or vasodilator iloprost in an adequate time frame may decrease the risk of amputation [12,23,24]. Nonetheless, more research is needed to evaluate the real impact of these therapies on frostbite sequelae. 

The rate of perceived exertion was higher in those patients who later underwent amputation. Although our results did not reach statistical significance, they match prior observations which considered that fatigue may predispose subjects to greater heat loss when exposed to cold [25]. 

Regarding the last, this theoretical prediction model for amputation risk presumes that below 7547 metres, the probability of amputation is lower than the possibility of preserving the affected appendages, as these values correspond to the negative values of the AI index. We must remark that our mathematical model has a certain specificity and sensibility that is dependent on our database content, and it is susceptible to improvement through the inclusion of more cases. 

The practical application of this mathematical model involves considering, in the medical advice previous to participation in an alpine expedition, which risks the alpinists face regarding sequelae from frostbite injuries at certain high altitudes. Taking into consideration that risk 0 does not exist, there are some points that can be considered as part of optimal management and prevention of sequelae from cold injuries at altitude (Table 2).

## 5. Conclusions

According to our observations, among all the considered variables, altitude is the best predictor of amputation rates after severe frostbite. Delays in rewarming the initial injury appeared to worsen prognosis, with a clear window of improved outcome opportunity during the first few hours. On the other hand, experience in winter mountaineering seems to be also a risk factor, probably due to the increased likelihood of taking part in more complex, technically demanding activities and at higher altitude, as well as the potential likelihood to assume more risks than less-experienced subjects. Altitude may be influenced by factors related to remoteness, because in most high-altitude expeditions the rescue times are prolonged and on-site or nearby medical assistance is usually limited.

Expedition members considering high- or extreme-altitude routes should consider carrying adequate and accessible supplies for easy and rapid rewarming and first aid of frostbite, in light of the importance of immediate management for the posterior evolution of frostbite. They should not rely only on their own experience, as simple exposure to cold seems to play a superlative role in the possibility of suffering frostbite. Regarding actual knowledge of advanced medical treatments, therapeutic time windows to hospital settings are short (<48 h) so the approximate rescue timing based on local and expedition resources should be known before travelling in order to facilitate decision making in the field and thereby to optimize prognosis.

## Figures and Tables

**Table 1:** Results for the prediction of amputation probability if frostbite occurs.

Cut-Off Point	True (%)	False (%)
0	100	0
0.05	100	15.09
0.1	90.48	39.62
0.15	80.95	58.49
0.2	80.95	58.49
0.25	80.95	62.26
0.3	80.95	64.15
0.35	80.95	64.15
0.4	71.43	75.47
0.45	61.9	84.91
0.5	52.38	88.68
**0.55**	**47.62**	**92.45**
0.6	9.52	100
0.65	0	100
0.7	0	100
0.75	0	100
0.8	0	100
0.85	0	100
0.9	0	100
0.95	0	100
1	0	100

**Table 2:** Suggested guidelines for the optimal management of high-altitude frostbite and the prevention of sequelae.

Guideline	Procedure	Evidence
**(1) Evaluate risk** for severe frostbite injuries with regard to ascent and team characteristics:	Consider the possibility of frostbite in spite of a high degree of expertise in winter mountaineering, regarding the level of exposure to the cold environment as a risk in itself.	Present study
	Consider the strong influence of altitude on amputation and sequelae (see Equation (1) AI in previous lines) if frostbite occurs.	Present study
	Consider the influence of the mountain range on amputation and sequelae, considering rescue timing to reference hospitals (Pyrenees/Alps < Himalayas < Karakoram).	Present study
	Consider the influence of logistics and the characteristics of each ascent (mountain, range, climbing style, etc.) leading to different complexities in providing prompt and adequate field treatment.	Present study
**(2) Minimize risks** of amputation and sequelae if frostbite occurs	Consider first-aid training for frostbite injuries as a must among all members of the expedition.	Hubell [26]
	Include in your fist aid kits those medications and resources needed in cases of frostbite.	Tek [27]
	Design and be aware of an evacuation schedule to first aid field installations, intermediate medical points (if they exist) and hospital or clinical settings, considering weather and local limitations for rescue.	Bowman and Kummerfeldt [28]
	Try to have effective communication with an expert in case you need advice or no medical staff are included in your expedition.	State of Alaska CIG [29]
**(3) Act correctly if frostbite occurs**	Learn to make correct and prompt identification of frostbite.	Zafren [5]
	Enact rapid rewarming if there is no reasonable possibility of secondary exposure to cold.	Syme [30]
	Provide optimal care for injuries and later treatment.	State of Alaska CIG [29]
**(4) Minimize time course** before receiving advanced treatments and medical advice if required	Provide the shortest evacuation time for severe injuries.	Linford et al. [31]
	Evacuate to a hospital where proper treatments can be administered.	State of Alaska CIG [29]
	Try to ensure correct management during rescue and transport.	State of Alaska CIG [29]
